# Transmission of waterborne parasites in the Association of Southeast Asian Nations (ASEAN): Overview and direction forward

**DOI:** 10.1016/j.fawpar.2017.08.001

**Published:** 2017-08-12

**Authors:** Yvonne A.L. Lim, Veeranoot Nissapatorn

**Affiliations:** a Department of Parasitology, Faculty of Medicine, University of Malaya, Kuala Lumpur, Malaysia; b School of Allied Health Sciences, Walailak University, Nakhon Si Thammarat, Thailand

**Keywords:** Waterborne, Parasites, Association of Southeast Asian Nations (ASEAN)

## Abstract

Most of the global outbreaks of waterborne parasitic protozoa have been reported in regions with established surveillance and reporting systems such as Australia, Europe, New Zealand, and North America. Given that only an estimated 1% of these outbreaks have occurred in Asia, it is evident that there is a paucity of information from this region where organised mechanisms of documentation of parasitic infections or waterborne outbreaks are lacking. This review attempts to provide an overview of the available data and studies on waterborne parasite occurrences among the Association of Southeast Asian Nations (ASEAN) which comprises of the ten member states (i.e., Brunei Darussalam, Cambodia, Indonesia, Lao People's Democratic Republic (PDR), Malaysia, Myanmar, the Philippines, Singapore, Thailand, and Vietnam) with the aims of identifying some directions on how to progress.

## Highlights

• 1% of global outbreaks of waterborne parasitic protozoa have been reported in Asia.
• Studies on occurrence of waterborne parasites are limited in ASEAN.
• Overview of the available data aims to identify the directions forward.

## 1 Global situation on waterborne parasites

In 2015, the World Health Organization estimated that 91% of the global population had access to an improved drinking water source through piped connection or other improved sources including public taps, protected wells and boreholes. Despite this achievement, at least 1.8 billion people are still using faecal contaminated drinking water sources which are causing an estimated 502,000 diarrhoeal deaths, each year (http://www.who.int/mediacentre/factsheets/fs391/en/; accessed 19/05/17). Diarrhoea remains the second major cause of death especially among children under 5 years of age (Kotloff et al., 2013, Checkley et al., 2015, Platts-Mills et al., 2015) and parasitic protozoa have been identified as one of the leading etiological agents of the diarrhoeal waterborne outbreaks.

The extent to which protozoan parasites contributed to global waterborne outbreaks between between early 1900s and December 2016 was reviewed. During this period spanning more than a century, a total of 905 outbreaks were documented worldwide [i.e., 325 outbreaks between early 1900 and 2004 (Karanis et al., 2007), 199 outbreaks between January 2004 and December 2010 (Baldursson and Karanis, 2011) and 381 outbreaks between January 2011 and December 2016 (Efstratiou et al., 2017)]. A majority of these outbreaks were reported in developed countries in North America and Europe, as well as in Australia and New Zealand. These countries had established surveillance and reporting systems which facilitate the dissemination of recorded data and detailed reports on waterborne parasitic protozoan outbreaks to the public (Efstratiou et al., 2017).

In contrast, limited outbreak data were recorded for developing countries, for example parts of Asia, Africa and Latin America. This was mainly due to the apparent lack of an organised and standardised documentation systems coupled with inadequately sensitive detection methods for protozoan infections or waterborne diseases, rather than it being a reflection of the actual scenario (Karanis et al., 2007, Baldursson and Karanis, 2011). In addition, there were no routine and coordinated efforts to collect epidemiological data on disease incidence attributed to protozoa and helminths. Moreover, data collection and reports on waterborne incidents or outbreaks besides these diseases not being regarded as notifiable diseases (e.g., Yoder et al., 2008).

In Asia, protozoan parasite outbreaks have been recorded in China (Feng et al., 2012, Wang et al., 2013), India (Palanisamy et al., 2006, Balasundaram et al., 2010), Japan (Ichinohe et al., 2005, Yokoi et al., 2005., Takagi et al., 2008) and Korea (Moon et al., 2013). In the review by Baldursson and Karanis (2011), it was highlighted that an outbreak incriminating parasitic protozoa such as *Giardia*, *Cryptosporidium*, *Entamoeba* and *Blastocystis* may have occurred in Malaysia. However, waterborne transmission of these parasites was not confirmed in this outbreak and, after taking into consideration all factors involved, the acute diarrhoeal outbreak was finally narrowed down to rotavirus as the causative agent (Hakim et al., 2007).

Although there have been a number of Malaysian studies on the occurrence of waterborne parasites (i.e., *Cryptosporidium*, *Giardia*, free living amoebae) (Lim and Ahmad, 2004a, Lim and Ahmad, 2004b, Lim et al., 2008, Onichandran et al., 2013, Lee et al., 2014, Abdul Majid et al., 2016, Kumar et al., 2014, Kumar et al., 2016), an outbreak implicating these parasites has not been documented thus far. Recent work by the Malaysian team and regional collaborators using a standardised method have also included analysis of water samples from surrounding countries such as the Philippines, Thailand, Vietnam (Kumar et al., 2016) and Lao PDR, Myanmar, and Singapore (Abdul Majid et al., 2016). These countries are part of the ten regional member countries of the Association of Southeast Asian Nations (ASEAN), comprising of Brunei Darussalam, Cambodia, Indonesia, Lao People's Democratic Republic (PDR), Malaysia, Myanmar, the Philippines, Singapore, Thailand, and Vietnam.

None of these countries has reported protozoa parasites as a cause of waterborne outbreaks. Given the geographical proximity of these countries and the paucity of available report on waterborne outbreaks in ASEAN, available epidemiological data and studies on waterborne occurrences in these countries are reviewed with the aim of identifying some directions on how to progress forward as a region.

## 2 Background on Association of Southeast Asian Nations

The Association of Southeast Asian Nations was founded in 1967 with the aims to forge cooperation in promoting economic and cultural development; trade, agricultural, industrial, and scientific collaboration; and peace and stability within the region (http://www.asean.org/; accessed 19/05/17). ASEAN covers a land area of approximately 3% of the total world land area and has a combined population of approximately 646 million people or almost 9% of the global population (http://www.worldometers.info/world-population/; accessed 19/05/17). Over the last 50 years since its inception, ASEAN has to some extent been successful in narrowing the development gaps and delivering more benefits to the people through substantial concerted effort. In 2015, with a collective total economy of USD2.4 trillion, ASEAN has been recognized as the sixth largest economy in the world and the third largest in Asia, potentially the next emerging market (http://asean.org/storage/2012/05/ASEAN_in_2016.pdf; accessed 19/05/17).

Despite these economic successes, economic disparities among the countries of ASEAN is still evident. Of the ten member countries, Brunei Darussalam and Singapore are among the richest; Indonesia, Malaysia, the Philippines and Thailand have robust economies; whilst Cambodia, Lao PDR, Myanmar and Vietnam are still developing (albeit varying success rates). Based on the Asia Water Watch (2015) report which projected based on past water supply coverage growth rates, countries in ASEAN are more likely to achieve the water supply coverage in the urban areas compared to rural areas (http://www.who.int/water_sanitation_health/publications/aww2.pdf; accessed 19/05/17). Given these discrepancies, it is crucial to evaluate how this could impact on the concerns of waterborne parasitic infections.

## 3 Current knowledge of waterborne parasites among ASEAN countries

Among the ASEAN countries, Malaysia has been actively collating information on waterborne parasites since 1997 till present. Other countries which have certain amount of accessible information include the Philippines, Thailand and Vietnam. More recently, there have been collaborative efforts to gather information simultaneously for Malaysia, the Philippines, Thailand, Vietnam, Lao PDR, Myanmar and Singapore. In contrast, no accessible data was found from Brunei Darussalam, Cambodia as well as Indonesia. A summary of key data on *Giardia* and *Cryptosporidium* in various water sample types in ASEAN nations is illustrated in Table 1.

**Table 1:** A summary of key data on *Giardia* and *Cryptosporidium* in various types of water samples in ASEAN countries.

Country^a^	Water type	Sample size	*Giardia* % positive	*Cryptosporidium* % positive	References
Malaysia	**Ground water**				
	Well water	28	17.9	7.1	Ahmad & Chan, 1994
	Well water	15	0	20	Ambu et al., 2014
	**Surface water**				
	River	174	39	11.5	Lim et al., 2008
	River	60	33.3	6.7	Azman et al., 2009
	River	39	51.3	23.1	Lee et al., 2014
	River	3	66.7	100	Kumar et al., 2014
	River	4	75	25	Ambu et al., 2014
	Lake	9	77.8	0	Lim et al., 2009a
	Lake (zoological)	18	94.4	0	Lim et al., 2009b
	Lake	13	69.2	61.5	Onichandran et al., 2013
	Lake	27	37.4	44.4	Kumar et al., 2014
	Lake	14	64.3	28.6	Abdul Majid et al., 2016
	Waterfall	3	100	33.3	Kumar et al., 2014
	**Recreational water**				
	Swimming pool	1	0	0	Kumar et al., 2014
	**Drinking water**				
	Tap water	5	0	0	Lim et al., 2008
	Tap water	2	0	0	Kumar et al., 2014
	Tap water	4	0	0	Ambu et al., 2014
	Mineral water	1	0	0	Kumar et al., 2014
	**Household water**				
	Household water stored in containers	20	10	0	Lim and Ahmad, 2004b
	**Drinking water treatment plant**				
	Raw water	87	46	6.9	Lim et al., 2008
	Treated water	87	0	0	Lim et al., 2008
	Backwash water	2	100	100	Lim et al., 2008
	Treatment Plant A	60	31.7	18.3	Richard et al., 2016
	Treatment Plant B	18	50	22.2	Richard et al., 2016
	Distribution system	7	0	14.3	Richard et al., 2016
	**Wastewater treatment plant**				
	Influent	30	100	40	Lim et al., 2008
	Effluent	30	83.3	20	Lim et al., 2008
Philippines	**Recreational water**				
	River	10	90	50	Onichandran et al., 2014
	Lake	6	66.7	33.3	Onichandran et al., 2014
	Pond	4	50	75	Onichandran et al., 2014
	Swimming pool	3	33.3	33.3	Onichandran et al., 2014
	Rain water tank	1	100	100	Onichandran et al., 2014
	**Drinking water**				
	Well	1	0	0	Onichandran et al., 2014
	Spring	1	0	0	Onichandran et al., 2014
	Tap water	3	0	0	Onichandran et al., 2014
	Tap water tank	1	0	0	Onichandran et al., 2014
	Water dispenser	2	0	0	Onichandran et al., 2014
	Mineral	1	0	0	Onichandran et al., 2014
Thailand	Bulk water for frozen food industry	20	60		Sutthikornchai et al., 2005
	Canal	120	98.3	75.8	Anceno et al., 2007
	Wastewater treatment plants	117	62.7	29.1	Anceno et al., 2007
	Rivers	52	50	25	Chuah et al., 2016
Vietnam	Vegetable splashing water	200	8.5	4.5	Tram and Dalsgaard, 2014

a Accessible data on *Giardia* and *Cryptosporidium* is not available from Brunei Darussalam, Cambodia, Indonesia, Lao PDR, Myanmar and Singapore.

### 3.1 Countries with information

#### 3.1.1 Malaysia

Based on accessible data among the ASEAN countries, Malaysia can be considered as one of the pioneer countries in the region in terms of waterborne parasites detection, in particular for *Cryptosporidium* and *Giardia*. Published data is available as early as 1997 on the occurrence of waterborne parasites in various water types (Ahmad et al., 1997). Prior studies focused on the detection of *Cryptosporidium* and *Giardia* due to global attention on the massive *Cryptosporidium* Milwaukee outbreak in 1993 which affected approximately 403,000 people and caused 104 deaths among the immunocompromised people (MacKenzie et al., 1994). Subsequently, studies also detected *Blastocystis*, free-living amoebae as well as other protozoans and helminths (Mat Amin et al., 2004, Ithoi et al., 2010, Ithoi et al., 2011, Kumar et al., 2014).

Methods used in Malaysia for the detection of *Cryptosporidium* and *Giardia* included large volume cartridge filtration (i.e., up to 50 L), membrane filtration (i.e., 10 L), elution and concentration of the eluate by centrifugation, immunomagnetisable particle separation and detection of (oo)cysts by epifluorescence microscopy according to USEPA Method 1623.1 (USEPA, 2012) with minor modifications.

Rivers

Based on a review on previous documented studies in Malaysia, 39% of 174 river water samples were positive for *Giardia* cysts (0.7–12,780 cysts per litre) and 11.5% for *Cryptosporidium* oocysts (0.4–246 oocysts per litre). This indicated a high level of contamination in rivers, which is worrying because some of these rivers are situated in catchment areas where water is sourced for drinking water (Lim et al., 2008). In the subsequent year, the occurrence of *Giardia* and *Cryptosporidium* (oo)cysts in two recreational rivers water was surveyed in Selangor state. The occurrence of both *Giardia duodenalis* and *Cryptosporidium parvum* (oo)cysts were 33.3% (20 of 60) and 6.7% (4 of 60), respectively (Azman et al., 2009). More recently, 39 river water samples were taken from rivers adjacent to five villages from three states in peninsular Malaysia (Lee et al., 2014). Of these, 51.3% samples were positive for *Giardia* cysts (0.10 to 25.80 cysts per litre) and 23.1% for *Cryptosporidium* oocysts (0.10 to 0.90 oocysts per litre). *Giardia* was detected in the river water samples from four villages whilst *Cryptosporidium* were detected in samples from two villages (Lee et al., 2014). All of these studies used the conventional microscopy method and viability testing was not included.

Besides *Cryptosporidium* and *Giardia*, a recent study determined the occurrence of *Blastocystis* sp. subtypes in river water and other water sources during wet and dry seasons around aboriginal villages. Water samples were collected from six sampling points of Sungai Krau and a point at Sungai Lompat and other water sources. Filtration of the water samples were carried out using a flatbed membrane filtration system and extracted DNA from concentrated water sediment was subjected to single round polymerase chain reaction and sequencing. Water samples collected from various water sources showed contaminations of *Blastocystis* sp. ST1, ST2, ST3 and ST4, during the wet season and *Blastocystis* sp. ST1, ST3, ST8 and ST10 during the dry season. *Blastocystis* sp. ST3 is suggested as the most robust and resistant subtype able to survive in any adverse environmental condition and commonly found in humans (Noradilah et al., 2016).

##### 3.1.1.1 Lakes

Two popular public recreational freshwater lakes were also investigated in Malaysia. Most of the 13 sampling sites at the lakes were contaminated with *Cryptosporidium* spp., *Giardia* spp., *Ascaris* spp. and hookworm (Onichandran et al., 2013). Another recreational lake situated in an urban area popular for its water-related activities was found to contain *Cryptosporidium*, *Giardia*, free-living amoeba, and helminth-like ova (Abdul Majid et al., 2016). Molecular tools were used in a study on recreational lake samples and 77.8% (of 9) of samples contained low numbers of *Giardia* cysts (range, 0.17–1.1 cysts/L). SSU rRNA gene sequencing analysis indicated the presence of *Giardia duodenalis* assemblage A suggesting potential risk to public health (Lim et al., 2009a). In another study, a combined microscopy-molecular approach was used to determine the assemblages of *Giardia* sp. cysts isolated from water samples collected from a zoological lake. *Giardia duodenalis* assemblage A and assemblage B, both infectious to humans, were identified at all sampling sites (Lim et al., 2009b).

##### 3.1.1.2 Wells

Many households in the northern state of Malaysia are dependent on well water for their daily chores and washing. A previous study found 17.9% and 7.1% of the well water samples were contaminated with *Giardia* cysts and *Cryptosporidium* oocysts, respectively (Ahmad & Chan, 1994). Upon further investigation, it was noted that some wells were located downstream of household toilets. The occurrence of *Giardia* and *Cryptosporidium* (oo)cysts in well water warrants further study especially in communities that remain dependent on wells for their water source, as do interventional and preventive steps, such as building toilets downstream of well locations and increasing hygiene awareness (Lim et al., 2008). More recently, well water samples from four sites (Sites A,B,C,D), a river and a tap inside a house were collected from a village in Selangor, which is the most developed state in Malaysia. The water samples were processed and examined for viruses, coliforms and protozoa as well as for heavy metal contaminants. The well-water was polluted with coliforms (1.2 to 2.4 × 10^3^ CFU/100 mL) in all sites, *E. coli* (0.12–4 × 10^2^ CFU/100 mL) and *Cryptosporidium* oocysts (0.4 cysts/100 mL). All the heavy metals and chemical parameters were within the Malaysian Guidelines' limits except manganese. These results highlighted that these well water sources are unsafe for direct consumption (Ambu et al., 2014).

##### 3.1.1.3 Drinking water treatment plants

In the late 1990s and early 2000, the quality of drinking water was evaluated in eight drinking water treatment plants. Although raw water supplying water treatment plants were highly contaminated with *Giardia* and *Cryptosporidium* (oo)cysts [concentration range: 0.03–120 (oo)cysts per litre], no (oo)cysts were detected in treated water (Ahmad et al., 1997, Tan, 1998, Lim and Ahmad, 2004a, Lim et al., 2008). The presence of (oo)cysts in raw water supplying water treatment plants and their absence in treated drinking water led to the postulation that (oo)cysts were accumulated in the treatment processes of the treatment plant. This was confirmed when two filter backwash water samples taken from a treatment plant were (oo)cyst positive [concentration range: 1200–2400 (oo)cysts per litre] (Lim et al., 2008). The accumulation of (oo)cysts in filters and backwash waters and the recycling of this backwash water has led to waterborne outbreaks in the UK, when oocysts accumulate in such numbers tend to overload the filtration capacity of the filter(s) and break through into drinking water (Anon, 1990).

As data was not available on the occurrence of waterborne parasites in drinking water at various processing sites of drinking water treatment plants (e.g., raw, coagulation, flocculation, sedimentation, filtration and treated water sites), a study was instigated in two major drinking water treatment plants (A and B) and seven distribution system (DS) sites in Sarawak, one of the largest states in Malaysia (Richard et al., 2016). Sampled water was positive for *Giardia* (32.9% of 85), *Cryptosporidium* (18.8%) followed by *Spirometra* ova-like (25.9%), *Blastocystis*-like (25.9%), nematode larvae-like (8.2%) and *Taenia* ova-like (1.2%). Meanwhile, 90.2% (of 61) samples were positive for *Acanthamoeba* and *Naegleria* via cultivation and of these, 11 isolates were confirmed as *Acanthamoeba* genotype T3 (5/7) and T4 (2/7) followed by *Naegleria* sp. (4/11), *Naegleria italica* (2/11), *Naegleria australiensis* (1/11), *Naegleria angularis* (1/11) *and Vahlkampfia* sp. (3/11). *Cryptosporidium*, *Acanthamoeba* and *Naegleria* were also detected in one of the seven tested DS sites. These parasites were detected frequently in raw water followed by the coagulation, flocculation, sedimentation and filtration sites (Richard et al., 2016).

##### 3.1.1.4 Post-treatment contamination

Whilst it is important to analyse the quality of treated drinking water, post-treatment contamination of drinking water with parasites is also crucial. In a study, *Giardia* cysts were found at 0.4 to 2 cysts per litre in the household water which was stored in a bucket (Lim and Ahmad, 2004b). The presence of cysts in drinking water is a public health risk to consumers of that source of water, because of the low infectious doses for cryptosporidiosis and giardiasis (9–1042 *C. parvum* oocysts and 10–100 *G. lamblia* cysts) (Rendtorff, 1954, Okhuysen et al., 1999). It was noted during sampling visits, that some families kept their water containers on the floor to make it convenient for young children to obtain water from them. Children with contaminated hands could have contaminated the water in the containers. As the prevalence of giardiasis in this community is high (19%) and was preponderant among children, the possibility of post-treatment contamination by children is very high (Lim et al., 1997).

Sewage treatment works (STWs)

Sewage treatment works (STWs) can also contribute (oo)cysts into receiving waters used for the abstraction of drinking water (Smith et al., 1995, Robertson et al., 1999, Robertson et al., 2000). In Malaysia, two studies have demonstrated that STWs can contribute *Giardia* cysts and *Cryptosporidium* oocysts to receiving waters used for the reclamation of drinking water (Lim, 1996, Lim et al., 2007). Concentrations ranged from 1 to 1462 cysts per litre for *Giardia* and 20–80 oocysts per litre for *Cryptosporidium* have been detected in the effluent. (Oo)cyst contaminated STW effluents can contaminate downstream water sources used by rural communities using river water as their main drinking water supply and services such as drinking water treatment works, livestock farms and food producers (Lim et al., 2007).

#### 3.1.2 The Philippines

In the Philippines, a total of thirty-three samples were analysed for the occurrences of waterborne parasites from various water samples (i.e., river, lake, pond, well, swimming pool, rain tank, water dispenser, tap, mineral water). These samples were processed to detect *Cryptosporidium* spp. *Giardia* spp., and free living amoebae, namely *Acanthamoeba* and *Naegleria* through microscopy examination and polymerase chain reaction (PCR) analysis. Of these, 36.4% of samples were positive for *Cryptosporidium* spp. (i.e., 5 river, 3 pond, 2 natural lake, 1 swimming pool and rain tank water samples each) whereas 45.5% samples were positive for *Giardia* spp. (i.e., 9 river, 4 natural lake, 2 pond, 1 swimming pool and rain tank water sample each). As for free-living amoebae, 33.3% samples were positive for *Acanthamoeba* spp. (3 river, 2 natural lake, pond, swimming pool and tap water sample each, 1 well and dispenser water sample each) whilst 18.2% samples were *Naegleria* spp. positive (1 river, natural lake, tap, dispenser and mineral water sample each) (Onichandran et al., 2014).

#### 3.1.3 Thailand

One of the first studies in Thailand to identify protozoa using specific monoclonal antibodies against *Giardia* and *Cryptosporidium* parasites followed by fluorescence microscopy in Thai frozen foods found *Giardia* cysts in 12 of 20 (60%) untreated water samples (60%) with an average of 53.33 cysts/1000 L. *Cryptosporidium* oocysts were detected in 7 samples (35%) with an average of 28.57 oocysts/1000 L. There were three samples of untreated water (15%) positive for both organisms. However, none of the treated water samples were contaminated. This study illustrated the potential for untreated water to contaminate food products and subsequently infect workers (Sutthikornchai et al., 2005). Two years later, a study by Anceno et al., 2007 showed that *C. parvum* oocysts and *G. duodenalis* cysts were present in 90% of two canals (i.e. Klong Neung and Klong Song) in Thailand. Molecular tools detected the presence of genotypes/assemblages of *C. parvum* and *G. duodenalis* in these canals suggested both human and animal sources of contamination. This result correlated with the pollution pressure originating from agricultural run-off upstream and populations in the surrounding area. Meanwhile, genotype 1 of *C. parvum* and both assemblages A and B of *G. duodenalis* were detected in the wastewater samples in which one of the canal passes through (Anceno et al., 2007. More recently, an investigation of the occurrence of *Cryptosporidium* and *Giardia* in the Kuang River Basin was carried out during the two distinctive dry and rainy seasons. *Giardia* was detected in 50% (26/52) of the river sampling sites while 25% (13/52) of these sites contained *Cryptosporidium* (Chuah et al., 2016).

*Cryptosporidium* and/or *Giardia* were detected in all the rivers with high prevalence in the upper Kuang River and Lai River. This is of significant concern as both drain into the Mae Kuang Reservoir, a vital source of drinking-water to many local towns and villages. During the dry season, *Cryptosporidium* or *Giardia* were detected in 21% (11 of 52) of the sampling sites and this rate doubled (40%; 21 of 52) during the wet season highlighting the significance of water as an agent of transport. With only basic water treatment facilities afforded to them, the communities of the rural area relying on these water supplies are highly at risk of acquiring *Cryptosporidium* and *Giardia* infections (Chuah et al., 2016).

Besides *Cryptosporidium* and *Giardia*, *Acanthamoeba* was also investigated in Thailand. Thammaratana et al. (2016) analysed 63 natural water samples from 11 provinces in northeast Thailand. The identification of *Acanthamoeba* was based on morphological features and molecular techniques using PCR and DNA sequencing. The results showed that 10 samples out of 63 were positive (15.9%). Phylogenetic analysis revealed that seven samples were T4, one sample was similar to T3, and the other two samples were similar to T5. Genotype T4 is considered the most commonly found in human keratitis and environmental isolates (Magnet et al., 2012) due to its greater virulence. This is the first report demonstrating the contamination in natural water sources in northeast Thailand (Thammaratana et al., 2016).

#### 3.1.4 Vietnam

In Vietnam, a study was conducted to evaluate a total of 200 water samples used by traders to moisten vegetables at markets located within eight districts in the city of Hanoi. These samples were analysed for the detection of faecal coliforms (*Escherichia coli*) and protozoan parasite (*Cryptosporidium* spp. and *Giardia* spp.). *Giardia* cysts were found in 17 (8.5%) splashing water samples and *Cryptosporidium* oocysts in nine samples (4.5%), with a median values of 20 cysts per ml and 10 oocysts per ml, respectively. *Escherichia coli* was found at a median concentration of 636 cfu per ml and its occurrence was negatively correlated with the numbers of protozoan parasites. The water for moistening vegetables was kept in buckets that were rarely cleaned and often used for handwashing. The finding of these pathogens in splashing water is likely to represent an important food safety hazards (Tram and Dalsgaard, 2014).

### 3.2 Regional collaborative efforts among ASEAN

More recently, there have been three regional collaborative efforts between Malaysia and Thailand (Kumar et al., 2014); among Malaysia, Philippines, Thailand and Vietnam (Kumar et al., 2016) and among Lao PDR, Myanmar, and Singapore (Abdul Majid et al., 2017) to collect current data using standardised method of sampling (membrane filtration), processing (concentration using IMS method) and epifluorescence microscopy.

#### 3.2.1 Malaysia and Thailand

This study investigated the distribution of parasites as the main contaminants in water environments of peninsular Malaysia (October 2011–December 2011) and the southeastern coast of Thailand (June 2012). Sixty-four water samples, 33 from Malaysia and 31 from Thailand, of various water types were examined according to U.S. Environmental Protection Agency guidelines. Drinking or household water types from both countries were free from parasitic contamination. The recreational/environmental (except a swimming pool in Malaysia) and effluent water types from these two countries were contaminated with waterborne parasites: *Giardia* (0.04–4 cysts/L), *Cryptosporidium* (0.06–2.33 oocysts/L), hookworm (6.67–350 ova/L), *Ascaris* (0.33–33.33 ova/L), and *Schistosoma* (9.25–13.33 ova/L). Higher concentrations of *Giardia, Cryptosporidium*, and hookworm were found in samples from Malaysia than in samples from Thailand. The presence of *Giardia* cysts showed a significant association (*p* < 0.005) with the presence of *Cryptosporidium* oocysts (Kumar et al., 2014).

#### 3.2.2 Malaysia, the Philippines, Thailand, Vietnam

The second collaborative effort involved Malaysia, Philippines, Thailand and Vietnam. This study was the first attempt aimed at detecting protozoan parasites in water samples from Southeast Asian countries, using real-time polymerase chain reaction (qPCR) assays. A total of 221 water samples of 10 L each were collected between April and October 2013 from Malaysia (53), Philippines (33), Thailand (120) and Vietnam (15). A physicochemical analysis was also conducted. The water samples were processed in accordance with the US Environmental Protection Agency's methods 1622/1623.1, microscopically observed and subsequently screened using qPCR assays. *Cryptosporidium* oocysts were detected in treated water samples from the Philippines (1/10), with a concentration of 0.06 ± 0.19 oocyst/L, and untreated water samples from Thailand (25/93), Malaysia (17/44), and the Philippines (11/23), with concentrations ranging from 0.13 ± 0.18 to 0.57 ± 1.41 oocyst/L. *Giardia* cysts were found in treated water samples from the Philippines with a concentration of 0.02 ± 0.06 cyst/L, and in untreated water samples from Thailand (20/93), Vietnam (5/10), Malaysia (22/44), and the Philippines (16/23), with concentrations ranging from 0.12 ± 0.3 to 8.90 ± 19.65 cyst/L. Species such as *C. parvum* and *G. lamblia* were detected using qPCR assays. *C. parvum* was detected in untreated water samples from the Philippines (1/23) and Malaysia (2/44), whilst, *G. lamblia* was detected in treated water samples from the Philippines (1/10) and in untreated water samples from Thailand (21/93), Malaysia (12/44), and the Philippines (17/23) (Kumar et al., 2016).

#### 3.2.3 Lao PDR, Myanmar and Singapore

The third effort was to gather information on the distribution of free-living amoebae (FLA). A total of 94 samples consisting of both treated and untreated water from Lao PDR (31), Myanmar (42), and Singapore (21) were investigated for the presence of pathogenic FLA. Each water sample was filtered and cultured onto non-nutrient agar seeded with live suspension of *Escherichia coli* and incubated at room temperature. Morphological identification was conducted for both trophozoites and cysts via microscopic stains (Giemsa and immunofluorescence). The presence of *Naegleria*-like structures was the most frequently encountered in both treated and untreated water samples, followed by *Acanthamoeba*-like and *Vermamoeba*-like features. To identify the pathogenic isolates, species-specific primer sets were applied for molecular identification of *Acanthamoeba, Naegleria*, and *Vermamoeba*. The pathogenic species of *Acanthamoeba lenticulata* and *A. triangularis* were detected from untreated water samples, while *Vermamoeba vermiformis* was found in both treated and untreated water samples. The results suggested that poor water quality as well as inadequate maintenance and treatment might be the cause of this alarming problem since chlorine disinfection is ineffective in eradicating these amoebas in treated water samples. Regular monitoring and water quality examination are necessary in order to control the growth, hence, further preventing the widespread of FLA infections among the public (Abdul Majid et al., 2017).

## 4 Gaps of knowledge

As described above, the reporting of waterborne parasites occurrence in the aquatic environment in many ASEAN countries has been sporadic except for Malaysia and more recently in Thailand and the Philippines. Currently, there is no available data from Brunei Darussalam, Cambodia or Indonesia. It is evident as shown in all studies that waterborne parasites are ubiquitous among ASEAN. For most countries, the available data has been infrequent, isolated and insufficient to provide a proper glimpse to generate an overall impression of the impact of waterborne route of transmission. Among the waterborne parasites, clearly, *Cryptosporidium* and *Giardia* are the most studied and significant to the global waterborne parasite transmission debate. Nonetheless, as observed in a number of these studies, waterborne contamination by other endemic parasites especially helminths and free living amoebae do occur as well.

While Malaysia is at the forefront of determining the occurrence and epidemiology of waterborne protozoan parasites, especially *Cryptosporidium*, *Giardia*, *Blastocystis* and free living amoebae among ASEAN countries, knowledge of helminth parasites occurrence and waterborne significance is still limited. Many rural or poorer areas in the ASEAN region are still plagued with helminth infections. Disregarding these parasites will be at our peril. The methods used to concentrate water for *Cryptosporidium* and *Giardia* analysis should also retain helminth transmission stages (i.e., ova). In the studies by Kumar et al. (2014) and Richards et al. (2016), concentrates were aliquoted for helminth ova detection. In many instances, the presence of oocysts and cysts in water concentrates collected from areas where human protozoan and helminth parasites are endemic, might act as an indicator for the presence of helminth parasites.

Many of the previous studies conducted used the conventional microscopy technique which has limitation in species determination. Consideration should be given to couple the usage of molecular tools with microscopy to enhance the information gathered in terms of differentiation of species and genotypes that are pathogenic versus non-pathogenic. In addition, viability estimation is equally crucial for the monitoring and control of these pathogens. Recently, a simple ‘one tube’ quantitative PCR (qPCR) protocol for viability estimation was developed using a ribosomal DNA marker (Paziewska-Harris et al., 2016). Studies which employed molecular techniques have shown to provide additional information that are beneficial in enhancing the understanding of the source of contamination (Lim et al., 2009a, Lim et al., 2009b, Kumar et al., 2016, Noradilah et al., 2016, Abdul Majid et al., 2017). Deciphering whether an organism is of human concern (i.e., a pathogen) and its viability are crucial as resources are limited among the ASEAN countries, therefore, efforts should be prioritised at targeting specific aims.

Currently, there is no consensus in the region on the adoption of microscopy methods developed by international agencies (e.g., USEPA) nor a standardisation of the molecular procedures. If such concerted effort is initiated within the region, it is crucial that the method adoption is adapted to local and regional environmental and geographical needs.

In addition, there continues to be a critical absence of reliable and established standardisation of surveillance and reporting systems for parasitic infections or waterborne outbreaks, whether nationally, regionally or globally. Currently, there is no organised practice that provides for identification let alone description and analysis of such events in this developing region. As a result, information regarding waterborne parasite outbreaks in developing countries is insufficient.

## 5 Moving forward as ASEAN region

Currently, the regulatory bodies in many countries in ASEAN do not monitor the presence of parasites in water used for consumption. Clearly, a regulatory or proficiency testing body is required to assure the quality of water with respect to parasites. Poor communities within the ASEAN nations, primarily rural, are at increased risk of not only waterborne protozoan pathogens but also other parasitic infections like helminths. This phenomenon retains them in the poverty trap and makes them less able to compete with their peers that drink water uncontaminated by parasites. For those who drink water from unimproved sources, the focus on optimising water treatment to remove protozoan parasites is mandatory to improve their overall standard of living.

As shown by the three regional effort studies (Kumar et al., 2014, Kumar et al., 2016, Abdul Majid et al., 2017), it is important for ASEAN countries to move forward in a concerted and collaborative approach, taking into consideration shared resources in terms of expertise, human resource, equipment and methodologies. The partnerships and network established among various countries during the above studies should be used as a platform to consolidate and to develop a framework for further regional collaboration. It is crucial to expand this partnership to other member countries.

The following is a proposed framework to propel this agenda forward.

• Assessment of existing methods/infrastructure for sanitation and water treatment in ASEAN nations.
• Development or adoption and adaptation of standardised and validated conventional microscopy and sensitive molecular methods for the isolation, purification and identification of parasites in water.
• Adoption of standardised and validated method to differentiate viable from non-viable parasites for detection and monitoring purposes. e.g. qPCR (Paziewska-Harris et al., 2016) and PMA-qPCR (Gyawali et al., 2016).
• Establishment of centralised national surveillance and reporting systems for parasites in particular *Cryptosporidium* and *Giardia*.
• Creation of coordinated national and regional databases on the occurrence of waterborne intestinal parasite for geographical and seasonal variations monitoring.
• Evaluation studies on recovery efficiency measurements and quantitative microbial risk assessment.
• Formulation of parasitological quality guidelines for a Southeast Asian edition of the Drinking Water Quality Guidelines.

## 6 Conclusions

Considering the high human population densities in many ASEAN countries, the limited or lack of access to safe drinking water, the pressures placed on water resources, and the increase in prevalence of diseases caused by diarrhoeal pathogens, waterborne parasites in particular, represent major and emerging threats to the health and well-being of people in the ASEAN. This is compounded by the fact that there are massive gaps of knowledge in the occurrence, morbidity and mortality associated with parasitic diseases.

Addressing the gaps in ASEAN countries will require sustained and substantial international efforts and investments. The above measures can be accomplished with the establishment of strong partnerships of researchers, public health and water quality professionals within the country and region. We must remember the clear benefits of developing sustainable access to safe drinking water and basic sanitation so that debilitating outcomes of parasitic infections and the poverty trap can be addressed.

## Conflict of interest statement

None of the authors has any financial or personal relationships that could inappropriately influence or bias the content of this paper.
